# Glutathione and Gts1p drive beneficial variability in the cadmium resistances of individual yeast cells

**DOI:** 10.1111/j.1365-2958.2007.05951.x

**Published:** 2007-11

**Authors:** Matthew C A Smith, Edward R Sumner, Simon V Avery

**Affiliations:** School of Biology, Institute of Genetics, University of Nottingham University Park, Nottingham NG7 2RD, UK

## Abstract

Phenotypic heterogeneity among individual cells within isogenic populations is widely documented, but its consequences are not well understood. Here, cell-to-cell variation in the stress resistance of *Saccharomyces cerevisiae*, particularly to cadmium, was revealed to depend on the antioxidant glutathione. Heterogeneity was decreased strikingly in *gsh1* mutants. Furthermore, cells sorted according to differing reduced-glutathione (GSH) contents exhibited differing stress resistances. The vacuolar GSH-conjugate pathway of detoxification was implicated in heterogeneous Cd resistance. Metabolic oscillations (ultradian rhythms) in yeast are known to modulate single-cell redox and GSH status. Gts1p stabilizes these oscillations and was found to be required for heterogeneous Cd and hydrogen-peroxide resistance, through the same pathway as Gsh1p. Expression of *GTS1* from a constitutive *tet*-regulated promoter suppressed oscillations and heterogeneity in GSH content, and resulted in decreased variation in stress resistance. This enabled manipulation of the degree of gene expression noise in cultures. It was shown that cells expressing Gts1p heterogeneously had a competitive advantage over more-homogeneous cell populations (with the same mean Gts1p expression), under continuous and fluctuating stress conditions. The results establish a novel molecular mechanism for single-cell heterogeneity, and demonstrate experimentally fitness advantages that depend on deterministic variation in gene expression within cell populations.

## Introduction

Individual genetically uniform cells within cell cultures exhibit marked phenotypic differences (i.e. heterogeneity), despite being of the same genotype. Such heterogeneity may be manifest in a wide range of phenotypes, many of which are fundamental to organism fitness and/or development. For example, individual cells of the same genotype may exhibit differing tendencies to differentiate and to express motility determinants, pathogenic cells may display variable degrees of virulence, and cells may have differing degrees of resistance to antimicrobial treatments and environmental stressors (reviewed in Avery, 2006). In addition, the principal control processes that regulate cell function (e.g. gene transcription, translation) at any moment in time may be differentially activated in different cells within genetically uniform populations. Recent studies have highlighted the contribution of stochasticity (noise) to such molecular-level variation (Kaern *et al*., 2005; Newman *et al*., 2006; Volfson *et al*., 2006; Struhl, 2007). Other potential drivers of heterogeneity involve epigenetic transitions in the state of the DNA molecule, and deterministic oscillatory changes in the physiological state of the cell (e.g. during the cell cycle) (Lloyd, 1993; Avery, 2006). There have now been a number of studies on gene expression noise in individual cells, and also on heterogeneity at the whole-cell phenotype level. However, very few studies have attempted to relate these aspects experimentally.

Exploiting the yeast model of heterogeneity, it was shown that cell cycle- and age-dependent regulation of the Cu,Zn-superoxide dismutase (Sod1) were key factors driving the variable Cu resistances of individual cells (Howlett and Avery, 1999; Sumner *et al*., 2003). It has now been established that Sod1p and other gene products can also act to suppress heterogeneity (in other phenotypes) among cells (Bishop *et al*., 2007). The latter study showed that enhanced phenotypic heterogeneity in certain mutants gave rise to increased rare-cell survival under severe stress. A specific advantage like this arising from heterogeneity touches on the other central question pertaining to heterogeneity: it has been hypothesized that cell populations benefit from phenotypic heterogeneity by the creation of subpopulations that could be better equipped to persist during perturbation and/or to exploit new niches (Tolker-Nielsen *et al*., 1998; Booth, 2002; Sumner and Avery, 2002). Consistent with such benefits, there is evidence of evolutionary selection for mechanisms generating phenotypic heterogeneity (True and Lindquist, 2000; Fraser *et al*., 2004; Raser and O'Shea, 2004). Furthermore, modelled simulations of competitions between heterogeneous and non-heterogeneous populations indicate that the heterogeneous populations could be more competitive, at least under certain conditions (Thattai and van Oudenaarden, 2004; Kussell and Leibler, 2005; Blake *et al*., 2006). Recently, Blake *et al*. (2006) also showed experimentally that phenotypic heterogeneity arising from the stochastic process of transcriptional bursting confers benefits. However, there remains a lack of such experimental evidence on benefits arising from heterogeneity, and this is particularly so for heterogeneity with a deterministic (i.e. non-stochastic) basis. An effective approach to addressing the latter central question would require an incisive means of manipulating the degree of deterministic heterogeneity expressed by cell populations. To date, this has been an elusive goal.

This study aimed to tackle both the questions of underlying molecular mechanism and of fitness consequences pertaining to heterogeneity. This required elucidation of a novel means to manipulate heterogeneity. Stress resistance provides a convenient binary output for measuring heterogeneity (Davey and Kell, 1996; Booth, 2002; Sumner and Avery, 2002). Heterogeneous resistance to oxidative stress is of particular interest, because of the well-documented role of reactive oxygen species (ROS) in disease aetiology (Halliwell and Gutteridge, 1999). Furthermore, ROS may be central to the modes of action of many stressors (Jamieson, 1998). Organisms have evolved enzymatic and non-enzymatic mechanisms for protection against oxidative stress. A major example of the latter is the essential metabolite glutathione, which provides reducing power for ROS scavenging (Wheeler and Grant, 2004). Glutathione also forms conjugates with xenobiotics such as cadmium for subsequent vacuolar detoxification (Li *et al*., 1997). Virtually all work to date on glutathione-dependent stress resistance phenotypes has relied on observations averaged across large numbers of cells, which mask effects at the single-cell level. However, it has been reported that the redox state of glutathione cycles in individual *Saccharomyces cerevisiae* cells during short-period, 40 min metabolic oscillations (ultradian rhythms) (Murray *et al*., 1999). Such oscillations become synchronized (and so recordable) during continuous yeast culture (Murray *et al*., 2003; Lloyd, 2006). Recent work has revealed that yeast metabolic oscillations are coupled to a periodicity in expression of different classes of genes and metabolites, extending across most of the genome and metabolome (Klevecz *et al*., 2004; Tu *et al*., 2005; Li and Klevecz, 2006; Murray *et al*., 2007). DNA synthesis in cells appears to be restricted to the reductive stage of the rhythms, which would limit the possibility of DNA damage from respiration-derived ROS (Chen *et al*., 2007). Furthermore, it has been reported that sensitivity to pro-oxidants such as menadione, H_2_O_2_ and cadmium fluctuates in continuous yeast cultures, with a similar periodicity as the ultradian oscillations (Wang *et al*., 2000; Tonozuka *et al*., 2001). In the present study, we observed marked cell-to-cell heterogeneity in glutathione-mediated stress resistance that was dependent on the *GTS1* gene product. *GTS1* transcription is known to modulate normal ultradian rhythmicity (Tonozuka *et al*., 2001; Adams *et al*., 2003). Therefore, manipulation of *GTS1* transcription provided a means to manipulate the degree of heterogeneity in yeast cultures, and so test the hypothesis that deterministic heterogeneity confers a fitness advantage. Such an advantage was demonstrated under both continuous and fluctuating stress conditions.

## Results

### Variation in GSH content determines phenotypic heterogeneity in cadmium resistance

**Fig. 1:**
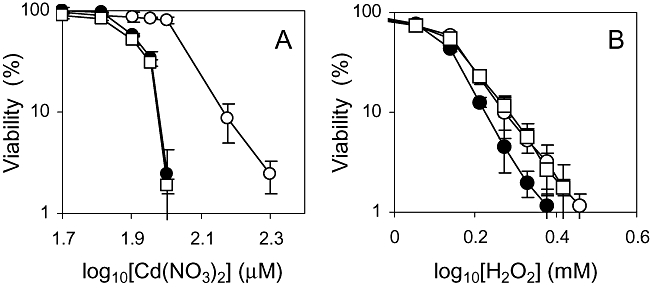
Influence of Gsh1p and Ycf1p on heterogeneous resistance to Cd and H_2_O_2_. Exponential-phase cultures of *S. cerevisiae* BY4743 (○) or isogenic deletion strains *gsh1*Δ (•) or *ycf1*Δ (□) were plated onto YPD supplemented with either Cd(NO_3_)_2_ (A) or H_2_O_2_ (B). Viability (colony formation) was determined after incubation for up to 8 days at 30°C, and converted to percentages by reference to growth on unsupplemented control medium. The points represent means from three replicate determinations ± SEM. Typical results from one of at least three independent experiments are shown.

**Fig. 4:**
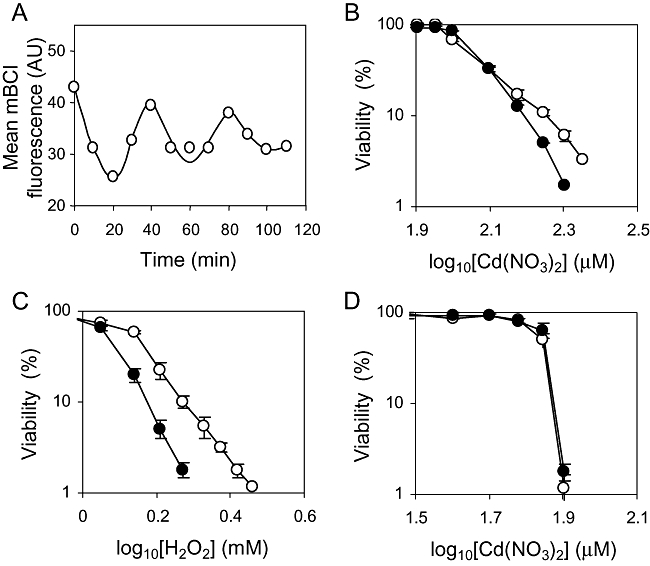
Gts1p, required for normal ultradian oscillations, is important for *GSH1*-dependent heterogeneity. A. A subpopulation comprising wild-type (BY4743) cells with the highest ∼20% mBCl fluorescences was gated and sorted. The mean mBCl fluorescence of this subpopulation was subsequently monitored with flow cytometry at intervals during shaking in YPD broth at 30°C. Each point represents data accumulated from 50 000 cells. Typical results from one of three independent experiments are shown. AU, arbitrary units of fluorescence. B and C. Exponential-phase cultures of *S. cerevisiae* BY4743 (○) or the isogenic deletion strain *gts1*Δ (•) were plated onto YPD supplemented with either Cd(NO_3_)_2_ (B) or H_2_O_2_ (C). D. Exponential-phase *gsh1*Δ (○) or *gsh1*Δ*,gts1*Δ (•) cells were plated onto YPD supplemented with Cd(NO_3_)_2_. Viability (colony formation) was determined after up to 8 day incubation at 30°C, and converted to percentages by reference to growth on unsupplemented control medium. The points represent means from three replicate determinations ± SEM. Typical results from one of several independent experiments are shown.

It was hypothesized that any variation in the cellular GSH contents of individual yeast cells could cause heterogeneity in GSH-dependent phenotypes. To test this, heterogeneity in a *gsh1*Δ-deletion strain (defective for the rate-limiting step of glutathione synthesis) was compared with that of wild-type cells. Heterogeneity was compared according to the relative gradients of dose–response curves, as described previously (Sumner *et al*., 2003; Bishop *et al*., 2007). Dose–response plots for the *gsh1*Δ mutant were shifted left relative to the wild type (Fig. 1). This indicated a culture-averaged sensitisation of *gsh1*Δ cells to Cd and H_2_O_2_, consistent with previous results (Wu and Moye-Rowley, 1994; Grant *et al*., 1996). Moreover, loss of Gsh1p was also associated with decreased cell-to-cell variability. Thus, loss of viability of mutant cells occurred over a narrower range of stressor doses (producing a steeper dose–response curve) than wild-type cells. An approximate 15-fold decline in viability of *gsh1*Δ cells (from 35% to 2.4%) resulted from only an ∼11% increase in Cd concentration (from 90 to 100 μM), whereas a similar loss of viability in wild-type cells required a > 70% increase in Cd concentration (Fig. 1A). No viable *gsh1*Δ cells could be detected at > 100 μM Cd. This degree of homogeneity resulting from a single gene knockout [equating to a heterogeneity ratio (HR) value ∼0.41; see *Experimental procedures*] was unprecedented in our experience, and was highly reproducible (further plots for wild-type and *gsh1*Δ cells are shown in Fig. 4B and D). GSH also contributed slightly to heterogeneous H_2_O_2_ resistance (Fig. 1B), but this effect (i.e. the difference in kill gradient for *gsh1*Δ versus wild-type cells) was considerably less marked than for Cd.

GSH-dependent Cd detoxification in yeast involves the formation of bis(glutathionato)cadmium (Cd·GS_2_) adducts (Li *et al*., 1997). These adducts are transported to the vacuole by Ycf1p. To see whether vacuolar uptake of Cd·GS_2_ (rather than adduct formation alone) was required to establish GSH-dependent heterogeneity, heterogeneous Cd resistance was also tested in a *ycf1*Δ mutant. Dose–response plots for the *gsh1*Δ and *ycf1*Δ mutants were superimposed (Fig. 1A). This indicated that the vacuolar detoxification pathway is the route through which heterogeneity is perpetrated. This was substantiated with a *vam1*Δ mutant which is defective for vacuole formation and maintenance. The *vam1*Δ mutant exhibited similarly decreased heterogeneity in Cd resistance (data not shown). As well as the Ycf1-mediated Cd detoxification mechanism, GSH also supplies reducing power for general antioxidant defence. This role is independent of Ycf1p. Accordingly, deletion of *YCF1* influenced neither culture-averaged nor heterogeneous H_2_O_2_ resistance (Fig. 1B). Because GSH could affect heterogeneity independently of Ycf1p (in the case of H_2_O_2_), the data suggested that the relationship between Ycf1p and heterogeneity seen with Cd (Fig. 1A) was more likely to be a consequence of heterogeneous GSH rather than heterogeneous Ycf1p levels.

**Fig. 2:**
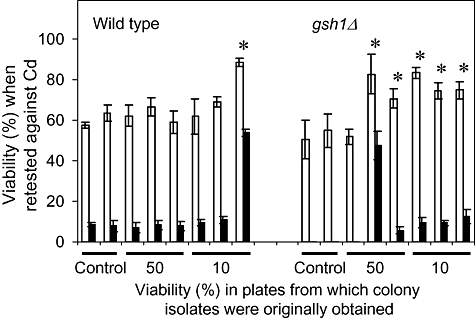
Single-cell Cd resistance in wild-type cells is non-inheritable. Exponential-phase wild-type (BY4743) or *gsh1*Δ cells were originally plated onto YPD supplemented with Cd(NO_3_)_2_ concentrations that gave either 100% viability (minus-Cd control), ∼50% viability (wild type, 150 μM Cd; *gsh1*Δ, 80 μM) or ∼10% viability (wild type, 200 μM Cd; *gsh1*Δ, 100 μM); viability being defined as colony-forming ability. Single-colony isolates from these plates (which in the case of the Cd-supplemented plates were Cd-resistant colonies) were subsequently inoculated directly in to liquid YPD medium in the absence of Cd(NO_3_)_2_ and, after 24 h exponential growth, % resistance to Cd was retested by plating aliquots of each isolate onto YPD agar supplemented or not with Cd(NO_3_)_2_. Data are shown for eight wild-type and eight *gsh1*Δ isolates, each obtained from plates that originally yielded the % viabilities indicated, and each retested for % resistance at the Cd concentrations which normally give ∼50% (□) or ∼10% viability (▪) of the relevant strains (see above for relevant concentrations). Percentage viabilities were calculated with reference to growth on minus-Cd control plates, and data are averaged from three replicate determinations ± SEM. Asterisks denote isolates exhibiting Cd resistance that is significantly higher (*P* < 0.05, according to Student's *t*-test) at both tested Cd concentrations than for control cultures not previously exposed to Cd.

To confirm that the single-cell phenotypes were not inheritable (i.e. not genotypic), a number of ‘resistant’ colonies isolated after an initial exposure to the stressor on agar were subcultured to YPD broth in the absence of stressor. These were grown for 24 h in the broth before resistance was retested (non-inheritable stress resistance is normally lost within 24 h of out-growth) (Bishop *et al*., 2007). Cadmium was selected for these tests because it yielded the strongest phenotype in *gsh1*Δ cells and because of its genotoxicity (Jin *et al*., 2003). This should enhance detection of any genotypic variation. The initial isolation of ‘resistant’ wild-type and *gsh1*Δ cells (via colony formation on Cd) was at Cd concentrations (see Fig. 2 legend) that gave either 100% viability (minus-Cd control), ∼50% viability or ∼10% viability. Resistant isolates subcultured from these plates (see above) were retested for resistance at each Cd concentration. Wild-type isolates that had not previously been exposed to Cd (‘control’ isolates) exhibited the anticipated ∼50% and ∼10% colony formation when tested at the relevant Cd concentrations (Fig. 2). With one exception out of six colonies tested, the same was true of cultures derived from Cd-resistant wild-type colonies (‘50’ and ‘10’ isolates in Fig. 2). This indicated that these cells did not retain their resistances during the intervening growth period in the absence of Cd. Therefore, the variable Cd resistances of wild-type cells was primarily due to phenotypic rather than genotypic (inheritable) heterogeneity. In contrast, occasional Cd resistance in *gsh1*Δ cultures appeared to be primarily genotypic rather than phenotypic: five out of six resistant isolates exhibited inheritable Cd resistance (Fig. 2) (note that the higher Cd concentration gave closer to 0% than the normal 10% viability in some *gsh1*Δ retests, reflecting the difficulty of reproducing equivalent dosages in successive experiments where the kill gradient is very steep; Fig. 1A). The results indicated that much of the residual detected variability in *gsh1*Δ cells was probably of genotypic origin. Therefore, the relative gradients shown in Fig. 1A actually underestimate the true impact of GSH on phenotypic (non-genotypic) heterogeneity. It is concluded that elimination of GSH synthesis eliminates most of the non-genotypic heterogeneity in Cd resistance, i.e. there is no GSH-independent mechanism that makes a substantial contribution to this heterogeneity phenotype.

**Fig. 3:**
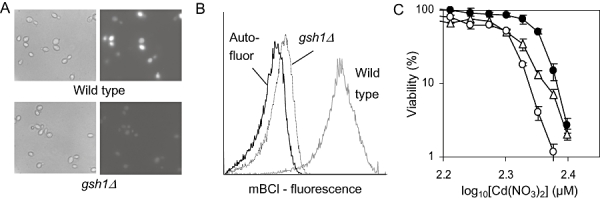
Cellular GSH content is variable and determines the Cd resistance of individual cells. Exponential-phase cells were stained with the GSH-specific fluorescent dye mBCl and analysed with fluorescence microscopy or flow cytometry. A. Phase-contrast (left panels) and fluorescence (right panels) images of mBCl-stained wild-type (BY4743) or *gsh1*Δ cells. B. Flow cytometric histograms of mBCl-fluorescence (on a logarithmic scale) in wild-type and *gsh1*Δ cultures; autofluorescence in unstained wild-type cells is also shown. C. mBCl-fluorescence histograms for wild-type cells (B) were gated and sorted into subpopulations containing cells with the lowest ∼20% (○) or highest ∼20% (•) fluorescences, or the entire cell population was gated and ‘sorted’ (▵). Sorted cells were plated onto YPD agar supplemented with Cd(NO_3_)_2_. Percentage viability was determined as colony-forming ability after 8 day growth at 30°C, with reference to control growth on Cd-unsupplemented agar. Bars represent means from at least three determinations ± SEM. Typical results from one of four independent experiments are shown.

The GSH-specific fluorescent dye monochlorobimane (mBCl) (Stevenson *et al*., 2002) was used to substantiate that the above effects on heterogeneity were due to single-cell GSH content. The specificity of mBCl for GSH was confirmed by the severe decrease in fluorescence in *gsh1*Δ cells versus wild-type cells (Fig. 3A). The intensity of mBCl fluorescence in individual wild-type cells varied markedly, and some cells exhibited negligible discernible fluorescence (Fig. 3A). This heterogeneity in GSH content was reflected by the dispersion of mBCl fluorescences among wild-type cells when analysed by flow cytometry (Fig. 3B). Note that in contrast to certain other forms of heterogeneity (Smits *et al*., 2006), we found no evidence for bistability (distinct subpopulations) in mBCl fluorescences among cells. Our flow cytometry data indicated a graded phenotype, and this correlated with the continuous gradients seen in dose–response curves. To verify that single-cell resistance was related to single-cell GSH content, wild-type cells were sorted into subpopulations comprising cells with the lowest ∼20% or highest ∼20% GSH contents (mBCl fluorescences). The sorted subpopulations were then tested for Cd resistance at a range of Cd concentrations (Fig. 3C). Percentage resistance to Cd was up to 10-fold greater in high-GSH cells than in low-GSH cells. The results show that single-cell GSH content is a key determinant of single-cell Cd resistance. Furthermore, the gradients of the plots (Fig. 3C) indicated that the degree of heterogeneity in the low- and high-GSH subpopulations was decreased markedly compared with that in a total-cell population (HR ∼0.57 and ∼0.49 respectively). This substantiated that the extent of heterogeneity in GSH content of cell populations determines the extent of heterogeneity in Cd resistance.

It should be emphasized that the results shown in Fig. 3C are with cells sorted according to their ‘basal’ GSH contents, i.e. before any exposure to Cd. Therefore, the results demonstrate that it is this basal GSH content that determines single-cell Cd resistance. This supports the hypothesis (Sumner and Avery, 2002; Bishop *et al*., 2007) that it is the initial (pre-induction) state of cells on first contact with stressor that is the major determinant of heterogeneous resistance in this type of experiment. Individual survivors may subsequently mount an adaptive response (e.g. *GSH1* induction in response to Cd) which should confer longer-term resistance and continued growth in the presence of stressor (Sumner and Avery, 2002), but this response does not determine the initial heterogeneity.

### Gts1p, required for normal ultradian oscillations, is important for *GSH1*-dependent heterogeneity

Levels of GSH are known to cycle during, and to help regulate, short-period metabolic oscillations in continuous cultures of *S. cerevisiae* (Murray *et al*., 1999). This could account for the cell-to-cell heterogeneity in GSH content. We confirmed such an oscillation among cells from the asynchronous batch populations used here. This involved sorting the high-GSH cells from cultures, to give a GSH-synchronized (homogeneous) subpopulation. Subsequently, changes in GSH content of these cells were monitored over time according to mBCl fluorescence. We observed a ∼40 min oscillation in GSH content, which was sustained for at least two cycles before the waveform became dampened as the subpopulation became less synchronous (Fig. 4A). This ∼40 min oscillation matched that observed elsewhere in oscillation-synchronized continuous cultures (e.g. Adams *et al*., 2003; Klevecz *et al*., 2004; Murray *et al*., 2007). The product of the *GTS1* gene is required for generating normal oscillations (Wang *et al*., 2001; Adams *et al*., 2003; Xu and Tsurugi, 2007), and Gts1p also binds to Ycf1p (Kawabata *et al*., 1999). Therefore, it was hypothesized that the GSH/Ycf1p-dependent heterogeneity in Cd and H_2_O_2_ resistance described above could be established by the Gts1p-dependent oscillatory behaviour of individual cells. Consistent with this, cells of a *gts1*Δ mutant were less heterogeneous than wild-type cells in their resistances to Cd and H_2_O_2_ (Fig. 4B and C). This effect was strongest for Cd, particularly when it is taken into account that the wild-type strain starts to decrease in viability at a slightly lower Cd dose than the mutant, which accentuates the heterogeneity difference between the strains (Fig. 4B). In the case of Cd, the effect of *GTS1* deletion on heterogeneity (HR ∼0.69) was less marked than that of *GSH1* (or *YCF1*) deletion (HR ∼0.41; Fig. 1A). In contrast, these genes had equivalent effects on heterogeneity in H_2_O_2_ resistance (HR ∼0.78 in both cases). Examination of heterogeneity in a *gsh1*Δ,*gts1*Δ double mutant revealed that the effects of *GSH1* and *GST1* deletion on heterogeneous Cd resistance were not additive (Fig. 4D). This suggests that the gene products modulate heterogeneity via the same pathway.

### *P*_*TET*_-regulated *GTS1* expression suppresses oscillations and heterogeneity in GSH content

**Fig. 5:**
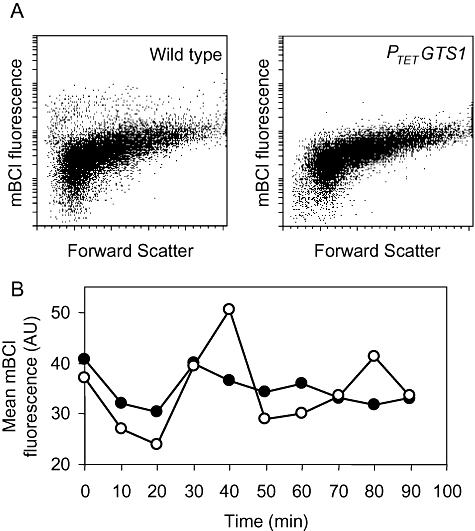
Oscillations and heterogeneity in cellular GSH content are suppressed in cells expressing *GTS1* under *P*_*TET*_ regulation. Exponential-phase cells were stained with mBCl and analysed by flow cytometry. A. The dot plots show fluorescence versus forward scatter properties of cells in cultures of wild-type cells (BY4743) and of cells in which the genomic *GTS1* promoter is replaced with *P*_*TET.*_ The doxycycline concentration (1 μg ml^−1^) used to regulate *P*_*TET*_ expression corresponded to culture-averaged expression of *GTS1* at ∼0.9× the level in wild-type cultures (see Fig. 6A). Similar results to those shown were obtained also at other doxycycline concentrations (data not shown). Each dot plot shows data accumulated from 20 000 cells. B. Subpopulations comprising cells with the highest ∼20% mBCl fluorescences from wild-type (○) or *P*_*TET*_*GTS1* (•) cultures were sorted and then incubated with shaking in doxycycline-supplemented YPD broth at 30°C. The mean mBCl fluorescences of the subpopulations were determined at intervals with flow cytometry. The doxycycline concentration (0.8 μg ml^−1^) used to regulate *P*_*TET*_ expression corresponded to culture-averaged expression of *GTS1* at ∼1.0× the level in wild-type cultures (see Fig. 6A). Each point represents data accumulated from 50 000 cells. AU, arbitrary units of fluorescence.

The influence of *GTS1* on metabolic oscillations in *S. cerevisiae* is known to be regulated at the level of *GTS1* transcription. Oscillations are markedly dampened when *GTS1* is expressed under the control of a constitutive promoter (Tonozuka *et al*., 2001; Wang *et al*., 2001). Consequently, we postulated that expressing *GTS1* behind an alternative promoter could provide a means to manipulate the degree of heterogeneity in stress resistance within cultures. This approach would avoid *GTS1* deletion, an important prerequisite for assigning a fitness effect specifically to heterogeneity (versus a culture-averaged change). For the same reason, we would need to express *GTS1* at a culture-averaged level equivalent to that in the wild type (albeit with altered heterogeneity). Therefore, we replaced the genomic *GTS1* promoter with the *tet-*regulated promoter system. This system gives constitutive gene expression at an averaged level that is scaleable with the concentration of doxycycline supplied (Belli *et al*., 1998a,b). To verify that *P*_*TET*_-regulated *GTS1* expression had the anticipated effects on heterogeneity, the constructed strain (*P_TET_GTS1*) was stained with mBCl and analysed for cell-to-cell variation. The GSH contents of individual cells were more homogeneous in *P*_*TET*_*GTS1* cultures (and in *gts1*Δ cultures; not shown) than in the wild type, as reflected by the narrower range of cellular mBCl fluorescences (Fig. 5A). The derived coefficients of variation (CVs) for mBCl fluorescence were 114.5 and 70.2 for the wild-type and *P*_*TET*_*GTS1* cultures respectively. In contrast, the culture-averaged GSH contents were similar. Mean mBCl fluorescences from independent replicates were 33.7 ± 0.9 and 35.1 ± 0.2 respectively. Based on the calculations of Drakulic *et al*. (2005), mean intracellular GSH was estimated as ∼10.4 and ∼10.8 mM respectively. A comparable dampening of heterogeneity in GSH content was observed also in *gts1Δ* cells with a *P*_*TET*_*GTS1* construct integrated at the *HO* site (data not shown). This suggested that the observed effects on heterogeneity were not dependent on genomic locus. The ∼40 min oscillation seen in sorted high-GSH cells of the wild type was strongly dampened in the *P*_*TET*_*GTS1* strain, which exhibited a more uniform GSH content over time after sorting (Fig. 5B). A similar dampening was seen in a *gts1*Δ mutant (data not shown). Therefore, the decreased heterogeneity in GSH content (Fig. 5A) could be attributed to suppression of Gts1p-dependent metabolic oscillations.

**Fig. 6:**
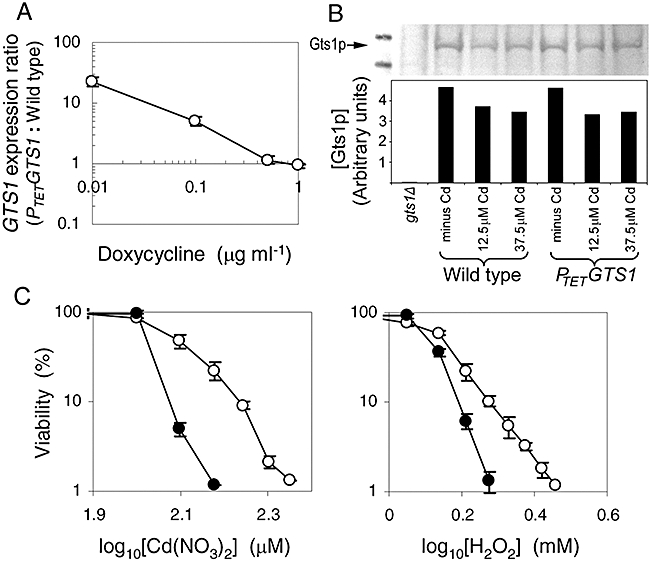
Homogeneous *GTS1* expression causes homogeneous stress resistance. A. *GTS1* expression (culture-averaged) in exponential-phase *P*_*TET*_*GTS1* cells was determined at varying doxcycline concentrations and plotted relative to *GTS1* expression measured in wild-type (BY4743) cells. *GTS1* mRNA in extracts was determined quantitatively by quantitative PCR in triplicate, with reference to *ACT1* mRNA. B. Western blotting was used to determine Gts1p levels in exponential-phase wild-type and *P*_*TET*_*GTS1* cells, incubated at 0.8 μg ml^−1^ doxycycline in either the absence or presence of 12.5 or 37.5 μM Cd(NO_3_)_2_. Each lane was loaded with protein extracted from 2 × 10^6^ cells [the decrease in Gts1p levels in Cd-treated cells is consistent with Cd-dependent inhibition of protein synthesis, as reported elsewhere (Lafaye *et al*., 2005); Cd has no effect on Gts1p as a proportion of total cellular protein (Vido *et al*., 2001)]. The lower panel shows densitometry analyses of Gts1p in each lane. The first lane contains protein markers. C. Exponential-phase cultures of wild-type (○) or *P*_*TET*_*GTS1* strains (•) were plated onto YPD supplemented with Cd(NO_3_)_2_ or H_2_O_2_ and 0.8 μg ml^−1^ doxycycline (to give equivalent culture-averaged *GTS1* expression; see A). Viability (colony formation) was determined after up to 8 days incubation at 30°C, and converted to percentages by reference to growth on unsupplemented control medium. The points represent means from three replicate determinations ± SEM. Typical results from one of at least three independent experiments are shown.

### Heterogeneous cell populations out-compete homogeneous cells during stress

To equalize mean Gts1p expression in wild-type and *P*_*TET*_*GTS1* cultures, *GTS1* transcript levels were measured with quantitative real-time polymerase chain reaction (qRT-PCR) over a range of doxycycline concentrations (Fig. 6A). As expected, the levels of *GTS1* mRNA in the *P*_*TET*_-regulated strain decreased relative to those in wild-type cells with increasing doxycycline concentration. *GTS1* expression was not affected by doxycycline in wild-type cells, consistent with observations elsewhere (Wishart *et al*., 2005). Doxycycline at 1.0 μg ml^−1^ gave a *GTS1* expression level that was just lower than that of wild-type cells (Fig. 6A), and no further repression of the *P*_*TET*_*GTS1* construct was observed at higher doxycycline concentrations. A concentration of 0.8 μg ml^−1^ doxycycline was selected for further experiments. This gives equivalent averaged-expression of *GTS1* in the two strains (Fig. 6A), but with altered consequences for heterogeneity (Fig. 5). We also employed Western blotting to confirm that the averaged level of the Gts1 protein was the same between the two strains at 0.8 μg ml^−1^ doxycycline, in either the absence or presence of Cd(NO_3_)_2_ (Fig. 6B). Moreover, GSH levels were also equalized between the strains, as described above (Fig. 5A).

Dose–response assays were used to test whether the effects of *P*_*TET*_*-*regulated *GTS1* expression on variability in cellular GSH content (Fig. 5) resulted in effects on heterogeneous stress resistance. This prediction was borne out, as the *P*_*TET*_*GTS1* strain exhibited decreased cell-to-cell heterogeneity (steeper dose–response plots) compared with the wild-type when tested over a range of Cd or H_2_O_2_ concentrations (Fig. 6C). This result was reflected by comparison of the Cd resistances of sorted high/low-GSH subpopulations (see Fig. 3) with the *P*_*TET*_*GTS1* strain. Whereas the wild-type subpopulations exhibited a > 50% difference in survival with Cd (Fig. 3C), equivalent tests with the corresponding *P*_*TET*_*GTS1* subpopulations yielded only a ∼30% survival difference (data not shown). Another key observation with the dose–response curves (Fig. 6C) was that the *P*_*TET*_*GTS1* plots were shifted left relative to the wild-type plots. This indicated culture-averaged sensitization to Cd and H_2_O_2_ in the *P*_*TET*_*GTS1* strain. Because culture-averaged *GTS1* expression and Gts1p levels (Fig. 6A and B), as well as mean GSH content (Fig. 5), were similar in the two cultures, it could be inferred that the advantage in wild-type cells was attributable specifically to heterogeneity.

**Fig. 7:**
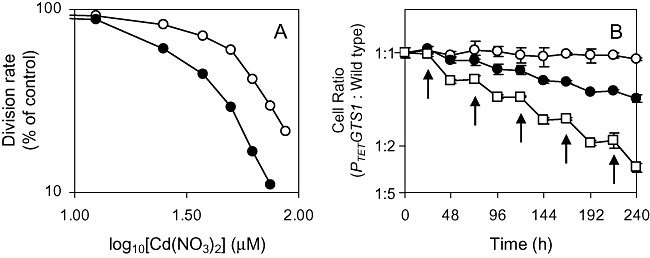
Heterogeneous cell populations out-compete homogeneous cells under Cd-stress conditions. A. Exponential-phase cells of the wild-type (BY4743) (○) or *P*_*TET*_*GTS1* (•) strains were incubated in YPD supplemented with the specified concentrations of Cd(NO_3_)_2_, with shaking in a microplate spectrophotometer (see *Experimental procedures*). All media were supplemented with 0.8 μg ml^−1^ doxycycline. Division rates (the number of generations per hour) were calculated during the exponential phase of growth ± Cd(NO_3_)_2_ and are expressed as the percentage of the control growth rate measured in the absence of Cd (the latter was the same in the two strains). Mean data were derived from two independent cultures of each strain (SEMs were smaller than the dimensions of the symbols), and typical data are shown from one of three independent experiments performed on different days. B. *P*_*TET*_*GTS1* and wild-type strains were co-cultured in YPD, starting from a cell density ratio adjusted to 1:1. Daily subcultures alternated between YPD medium and either further YPD (○), or YPD + 12.5 μM Cd(NO_3_)_2_ (•), or YPD + 37.5 μM Cd(NO_3_)_2_ (□). All media were supplemented with 0.8 μg ml^−1^ doxycycline. The relative cell numbers of the two strains were determined by plating culture aliquots on YPD agar ± G418 at the end of each 24 h growth period in the absence (preceding arrow) or presence (following arrow) of Cd(NO_3_)_2_. Colony-forming units (cfus) were enumerated after 3 days incubation at 30°C; *P*_*TET*_*GTS1* numbers were determined from the +G418 plates, and wild type from the difference between that number and the cfus on YPD. Points represent means of ratios ± SEM determined from two independent experiments, each consisting of three replicate cfu determinations at each time point.

To test this inference more rigorously, the cell division rates of the *P*_*TET*_*GTS1* and wild-type strains during exponential-phase growth in broth were determined over a range of Cd concentrations. The two strains exhibited very similar growth in the absence of Cd and at non-inhibitory Cd concentrations (< 12.5 μM Cd). However, the inhibitory effect of higher Cd concentrations on relative division rate was more marked in *P*_*TET*_*GTS1* than in wild-type cultures (Fig. 7A). Thus, at 75 μM Cd(NO_3_)_2_ the division rate of the wild type was still ∼30% of that measured in the absence of Cd, whereas the corresponding determination for the *P*_*TET*_*GTS1* strain was only ∼11%.

The hypothesis was tested further in direct competition assays. These were conducted under alternating control and Cd-stress conditions. This was because it has been speculated that non-genotypic heterogeneity might be particularly important in allowing survival of intermittent environmental stresses, in which a subset of resistant cells might survive and then re-establish populations under less stressful conditions (Tolker-Nielsen *et al*., 1998; Avery, 2006). The *P*_*TET*_*GTS1* and wild-type strains were initially mixed in 1:1 ratios and then co-cultured for 10 days, with daily subculture to maintain exponential phase. The test incubations comprised 24 h without Cd, then 24 h with Cd, over five cycles. The fitness-neutral *KanMX4* marker in *P*_*TET*_*GTS1* cells was exploited to discriminate the strains for enumeration on agar, with or without selection by G418. Control incubations, with daily subculture in the absence of Cd throughout, confirmed that neither strain had a competitive advantage over the other under non-stress conditions (Fig. 7B). This was evident also from the control growth periods (preceding the arrows) in the alternating control/Cd incubations, during which the strain ratios did not change (Fig. 7B). In contrast, each 24 h growth period in the presence of Cd was associated with a marked shift in the relative proportions of the two strains, favouring the wild type. After five growth cycles ± 37.5 μM Cd, the ratio of *P*_*TET*_*GTS1* to wild-type cells had shifted from 1:1 to 1:3. Consistent with the previous data (Fig. 6A and B), semiquantitative RT-PCR as well as Western blot analyses confirmed that culture-averaged Gts1p expression did not differ significantly between the two strains (these analyses were with the strains growing separately under the same conditions as used in the main experiment, either in the absence or in the presence of Cd). Thus, out-competition of *P*_*TET*_*GTS1* cells by wild-type cells during stress can be linked to the enhanced phenotypic heterogeneity of the wild-type cells.

## Discussion

The results presented here reveal a novel deterministic mechanism by which cells generate heterogeneity. In addition, that insight is exploited in demonstrating experimentally that such heterogeneity confers a fitness advantage in cell populations. It is of particular interest that heterogeneity was attributable to cell-to-cell variation in GSH content. Glutathione, an essential metabolite, affects a broad range of cellular phenotypes (Wheeler and Grant, 2004). The influence of GSH seems likely to extend also to heterogeneity in many of those phenotypes. However, interestingly, deletion of *GSH1* was found previously to have no significant influence on heterogeneous resistance to copper (Sumner *et al*., 2003). That former study used a shorter-term assay of heterogeneity than the colony-growth assay used here, the latter offering higher reproducibility. Nonetheless, we have shown how the nature of the heterogeneity assay has little influence qualitatively on outcome, relative to the gene–phenotype combination being studied (Bishop *et al*., 2007). Therefore, this difference between the studies appears to relate to the different types of metals examined. Consequently, the heterogeneity effects described here with Cd and, to a lesser extent, H_2_O_2_ should not necessarily be presumed to extend to any of the stressors that are known to be potentially influenced by GSH (Wheeler and Grant, 2004).

Deletion of *GSH1* almost eliminated heterogeneity in Cd resistance here. As a consequence, relatively small increments of increasing Cd concentration were required to be able to observe intermediate levels of killing (i.e. other than 0% or 100%) in *gsh1*Δ cells. The residual heterogeneity in *gsh1Δ* cells appeared to be of genotypic rather than non-genotypic origin, according to inheritability experiments. It cannot be discounted that there was also some epigenetic component to the inheritability (Xu *et al*., 2006), although the fact that the influence of *GTS1* on heterogeneity was not affected by expression from an alternative genomic locus (*HO*) provides one argument against locus-dependent epigenetic regulation. Moreover, the degree of *homo*geneity accomplished here is unique in our experience. This underscores not only the importance of GSH in driving heterogeneous Cd resistance, but also the potential value of this system as a broader model of population homogeneity.

Although not redox active, like several other metals Cd is thought to have an oxidative mode of toxicity. Thus, Cd-induced lipid peroxidation causes membrane permeabilization and loss of viability, cellular thiols become depleted during Cd exposure, and the cellular response to Cd stress has marked overlaps with that to oxidative (especially peroxide) stresses (Howlett and Avery, 1997; Lee *et al*., 1999; Avery, 2001; Avery *et al*., 2004). Nevertheless, GSH-dependent detoxification of Cd differs from that of H_2_O_2_, in that only the former involves vacuolar sequestration via Ycf1p (although Ycf1-mediated peroxide detoxification has been reported in glutaredoxin-overexpressing cells; Collinson *et al*., 2002). This difference in the action of GSH against Cd and H_2_O_2_ could explain why *GSH1* deletion had a greater effect on heterogeneity in Cd resistance than in H_2_O_2_ resistance in this study, i.e. the GSH-Ycf1p pathway might make a more important contribution to phenotypic ‘noise’ than the GSH-dependent mechanisms involved in H_2_O_2_ detoxification (e.g. supply of reducing equivalents to peroxidases). In keeping with this suggestion, it is known that the Gts1 protein – which was shown here to help establish GSH-dependent heterogeneity, according to epistasis tests (Fig. 4D) and flow cytometry-based assays (Fig. 5) – additionally binds Ycf1p, with potential consequences for Ycf1 activity (Kawabata *et al*., 1999). If Ycf1p function as well as GSH content fluctuates according to single-cell Gts1p activity, this could serve to amplify ‘noise’ in the GSH-Ycf1p pathway of Cd resistance. Noise amplification mechanisms have been described in other biochemical systems (Samoilov *et al*., 2005). On the other hand, *GSH1* (or *YCF1*) deletion had a greater effect on heterogeneity than *GTS1* deletion in the case of Cd resistance, but not in the case of H_2_O_2_ resistance (see Figs 1 and 4). One interpretation of this result is that the specific Gts1p–Ycf1p interaction, which is relevant only to Cd, could in fact help to stabilize heterogeneity. This alternative scenario would partly counterbalance the redox oscillation-associated heterogeneity within the GSH-Ycf1p pathway (also affected by Gts1p) (Murray *et al*., 1999), potentially offering a mechanism for fine-tuning the degree of culture heterogeneity. Further investigation of such possibilities will require a better understanding of the functional significance of the Gts1p–Ycf1p interaction (Kawabata *et al*., 1999), which has not yet been elucidated.

The role of Gts1p in the heterogeneity described here adds further insight to this protein's function. Gts1p was originally considered a putative clock protein and essential for yeast metabolic oscillations (Mitsui *et al*., 1994; Wang *et al*., 2001). However, more recent work has indicated that oscillations can persist in *gts1*Δ mutants, albeit with shortened phase and decreased stability (Adams *et al*., 2003). Similarly, oscillations were dampened by *P*_*TET*_-regulated constitutive expression of the *GST1* open reading frame (ORF) in the present study. The collective evidence indicates that Gts1p function interfaces with the central oscillating loop and is a key regulator of ultradian rhythms in yeast (Jules *et al*., 2005; Xu and Tsurugi, 2007). These oscillations drive genome-wide fluctuations in gene expression (Klevecz *et al*., 2004; Tu *et al*., 2005; Li and Klevecz, 2006). However, their study to date has been restricted to continuous cultures or cell cycle-synchronized batch cultures, in which the oscillations become synchronized across cell populations. The single-cell and sorting assays exploited here have demonstrated the importance of Gts1p-dependent variation also within asynchronous batch cultures, and so offer the opportunity to extend study of these oscillations to such heterogeneous populations.

The influence of *GTS1* on yeast metabolic oscillations is regulated at the level of *GTS1* transcription (Tonozuka *et al*., 2001; Wang *et al*., 2001). This was exploited here to manipulate heterogeneity. Through the use of *P*_*TET*_, we achieved our aim of maintaining culture-averaged *GTS1* expression and GSH content while suppressing cell-to-cell heterogeneity (by approximately 40% according to relative CV values for population mBCl fluorescence). Thus, comparison of wild-type (heterogeneous) cells with *P*_*TET*_*GTS1* (homogeneous) cells provided a novel model system for dissecting the impact specifically of heterogeneity on culture fitness. Such an impact became evident during stress, as the heterogeneous population out-competed the homogeneous population in three different experimental assays, despite identical growth rates under non-stress conditions. The latter observation indicates that generation of heterogeneity has no metabolic cost at the population level, at least in the example studied here.

The results provide direct experimental evidence to support the increasingly suggested hypothesis that phenotypic heterogeneity confers fitness advantages in cell populations during stress (Conrad, 1977; Tolker-Nielsen *et al*., 1998; Sumner and Avery, 2002; Thattai and van Oudenaarden, 2004; Kaern *et al*., 2005; Avery, 2006). It can be inferred that Gts1p-regulated redox oscillations in wild-type cultures generate cells with varying GSH contents, as suggested previously (Murray *et al*., 1999). Moreover, we show that the cells with higher than average GSH contents have an advantage in the face of H_2_O_2_ or Cd stress. Such cells could be critical for the persistence of the organism during stresses that kill large numbers of cells in a population.

The advantage of heterogeneity was evident here even during non-lethal (but growth-slowing) Cd stress (see Fig. 7A). These growth data indicated that the relationship between single-cell GSH content and Cd resistance is not linear: such a relationship would be expected to yield similar culture-averaged growth rates between populations under Cd stress when, as was the case here, culture-averaged GSH content is similar. Rather, the results are consistent with a threshold Gts1p or GSH level (achieved only in a fraction of wild-type cells, and fewer, if any, *P*_*TET*_*GTS1* cells) above which Cd resistance is elevated markedly, leading to faster net population growth. Similar threshold effects on the outcomes from heterogeneity have been described elsewhere (Blake *et al*., 2006). The stress-specific advantage of wild-type cells was also marked in competitions under alternating Cd-stress and non-stress conditions. This is the type of situation in which phenotypic heterogeneity has been considered likely to be particularly advantageous (Thattai and van Oudenaarden, 2004; Kaern *et al*., 2005; Avery, 2006). However, our long-term agar growth assays reveal that intervening growth periods without stress are not a prerequisite for such advantages.

It should be emphasized that disadvantages of homogeneity may not be apparent when a less-heterogeneous subpopulation has an alternative advantage, such as enhanced mean GSH. This was illustrated in the sorting experiments where sorted high-GSH cells had higher Cd resistance than the total population, despite lower heterogeneity (Fig. 3C). Such effects can be controlled against experimentally by equalizing mean expression between test and control cultures, as we did here with the *P*_*TET*_*-GTS1* and wild-type strain comparisons.

In conclusion, recent microarray studies have led to the proposal that ultradian rhythmicity in yeast serves to separate temporally cellular processes that may be incompatible, such as respiration (with associated generation of ROS) and the restructuring of chromatin to enable DNA replication. The present study reveals another outcome of Gts1p-dependent rhythmicity: the generation of heterogeneity which is beneficial to cell populations. This demonstration of a benefit from deterministic variation among cells completes the picture initiated by a recent study of phenotypic variation that is of stochastic origin (Blake *et al*., 2006). Therefore, this work has elucidated a connection between short-period biological rhythmicity and cell individuality. It also provides new experimental evidence to help explain why these processes may have evolved in cell populations.

## Experimental procedures

### General culture conditions

Yeast strains were maintained and grown in YPD broth [2% (w/v) bacteriological peptone (Oxoid), 1% yeast extract (Oxoid), 2% d-glucose] or in YNB medium [0.69% yeast nitrogen base without amino acids (Formedium), 2% (w/v) d-glucose] supplemented as required with amino acids or uracil (Ausubel *et al*., 2007). When selection was needed, either hygromycin B (Invitrogen) or G418 (Sigma) was added to media to a final concentration of 250 μg l^−1^. Where necessary, media were solidified with 2% (w/v) agar (Sigma). Experimental *S. cerevisiae* cultures were inoculated from overnight starter cultures grown from single colonies, and cultured to exponential phase (OD_600_∼2.0) in liquid medium at 30°C, 120 r.p.m.

### Yeast strains and DNA manipulations

*Saccharomyces cerevisiae* strains BY4741 (*MAT***a**; *his3*Δ1; *leu2*Δ0; *met15*Δ0; *ura3*Δ0), BY4742 (*MAT***α**; *his3*Δ1; *leu2*Δ0; *lys2*Δ0; *ura3*Δ0), BY4743 (*MAT***a**/**α**; *his3*Δ1/*his3*Δ1; *leu2*Δ0/*leu2*Δ0; *lys2*Δ0/*LYS2*; *MET15*/*met15*Δ0; *ura3*Δ0/*ura3*Δ0) and derivative single deletion mutants were obtained from Euroscarf (Frankfurt, Germany). A *gts1*Δ*,gsh1*Δ double deletion strain in the BY4743 background was created after disruption of *GSH1* in *gts1*Δ single mutants from each of the haploid BY4741 and BY4742 backgrounds, using the *HphNT1* cassette (Janke *et al*., 2004) to replace the entire *GSH1* ORF by short flanking homology (SFH) PCR (Wach *et al*., 1997). Transformation was by the lithium acetate method (Gietz and Woods, 2002), and appropriate integration of the cassette was confirmed by diagnostic PCR (Wach *et al*., 1997). The haploid double mutants were mated by co-incubation overnight on YPD agar without selection, followed by two washes in sterile distilled water and incubation for 5 days on YNB agar supplemented with appropriate amino acids for selection.

To construct a cassette giving *tet*-regulated *GTS1* expression, plasmid pCM225 containing *P*_*TET*_ (consisting of *KanMX4* as the selectable marker, the tetracycline-responsive tTA activator gene and the *tetO*_7_ promoter) (Belli *et al*., 1998a) was cut with PvuII and BglII, recessed DNA ends were filled with Klenow and ligated to produce plasmid pMS01. A 1.2 kb PCR fragment containing the *GTS1* ORF was amplified from yeast genomic DNA with addition of terminal BclI and HpaI sites, and cloned between the BamHI and HpaI sites of pMS01 to yield plasmid pMS02 containing *P*_*TET*_*GTS1*. A 1 kb region upstream of the *GTS1* ORF (and preceding the *GTS1*pr.183 sequence required for oscillatory *GTS1* transcription) (Tonozuka *et al*., 2001) was amplified using primers which incorporated terminal NotI and BstEII restriction sites and ligated between the corresponding sites in pMS02. The resulting plasmid, pMS06, was digested with NotI and SfoI to release a 6.2 kb cassette containing *P*_*TET*_*GTS1* and targeted for integration into the genome at the native *GTS1* locus via the cloned 1 kb upstream fragment and the *GTS1* ORF. This *P*_*TET*_*GTS1* cassette was transformed into BY4741 and BY4742 cells, G418-resistant transformants were screened for appropriate integration and haploid strains mated as described above to create the diploid *P*_*TET*_*GTS1* strain. To produce an equivalent cassette targeted to the *HO* locus, pMS02 was cut with NotI and SapI and recessed ends filled with Klenow to generate a blunt *KanMX4*, *P*_*TET*_*GTS1* fragment. This fragment was ligated into pBSTHO at the Klenow-filled EcoRI site, which lies in between ∼500 bp regions cloned from the 5′ and 3′ ends of the *S. cerevisiae HO* ORF (Payne, 2006). The product, pMS04, was digested with NotI and AarI to generate a 6.4 kb *P*_*TET*_*GTS1.HO* cassette, which was transformed into BY4741 and BY4742 cells, in which the *GTS1* ORF had previously been deleted by SFH PCR using the *HphNT1* cassette, as described above. The resultant strains were mated to create a diploid *P*_*TET*_*GTS1.HO* strain. All DNA cloning and genetic manipulations were performed in *Escherichia coli* strain DH5α (Invitrogen). Restriction digests, DNA ligations, sequencing and PCR were carried using standard protocols (Ausubel *et al*., 2007). All primer sequences are available upon request.

### Dose–response curves

Exponential-phase experimental cultures (see above) were diluted in fresh YPD, and cells (∼200 per plate) were spread plated on YPD agar supplemented or not with the specified concentrations of Cd(NO_3_)_2_ or H_2_O_2_. All media for experiments involving *P*_*TET*_-regulated *GTS1* expression additionally were supplemented with 0.8 μg ml^−1^ doxycycline. Colonies were enumerated after incubation at 30°C for up to 8 days, to allow for any slow growth under the stress conditions. Percentage resistances were calculated with reference to control incubations in the absence of stressor. HRs were calculated according to Sumner *et al*. (2003), to provide a measure of mutant versus wild-type gradients (heterogeneity) in dose–response curves. (In the sorting experiment of Fig. 3, HR was calculated from comparison of sorted versus unsorted subpopulations.) HR was defined as the ratio of the log increases in stressor concentrations required to give 1-log decreases in viability for mutant cultures relative to wild-type cultures (we used a 50% to 5% decrease in viability as a convenient 1-log standard). Complete elimination of heterogeneity would give an HR value of 0, whereas HR ∼1.0 would indicate that the deleted gene has no effect on heterogeneity.

### Growth rate determinations and direct competition assays

Growth rates for *P*_*TET*_*GTS1* and BY4743 strains (indirect competitions) were determined separately using a BioTek Powerwave XS microplate spectrophotometer. Experimental cultures (OD_600_∼2.0) grown in YPD containing doxycycline at 0.8 μg ml^−1^ were diluted (OD_600_∼0.1) into pre-warmed fresh medium and 300 μl samples aliquoted into 48-well plates (Greiner Bio-One) before addition or not of Cd(NO_3_)_2_ at the specified concentrations. Cultures were incubated at 30°C with shaking for 40 h, with the OD_600_ measured every 30 min. Cell division rates (the number of generations per hour) in the presence of Cd(NO_3_)_2_ were derived from a minimum of at least four OD_600_ determinations obtained during exponential growth, and were calculated as a percentage of the control growth rate measured in the absence of Cd(NO_3_)_2_.

Direct competition assays were performed by mixing exponential-phase *P*_*TET*_*GTS1* and BY4743 strains in a 1:1 ratio (based on OD_600_) into fresh YPD supplemented with 0.8 μg ml^−1^ doxycycline, and Cd(NO_3_)_2_ as required. Cultures were grown for 10 consecutive 24 h periods at 30°C, 120 r.p.m., with daily subculture to maintain exponential growth and allow media switches. At the time of each subculture, samples of cells were diluted in YPD before spread plating (∼200 cells per plate) on YPD agar supplemented or not with G418. Colonies were enumerated after incubation at 30°C for 3 days. The ratios of *P*_*TET*_*GTS1* to wild-type cells in cultures were calculated by comparison of the numbers of G418-resistant (*P*_*TET*_*GTS1*) and G418-sensitive (wild type) colonies.

### Determination of *GTS1* transcript levels

RNA was extracted from cells using the hot acid phenol method (Schmitt *et al*., 1990). Residual DNA was removed by treatment with RNase-free DNase (Promega), and treated RNA was recovered using an RNeasy Mini kit (Qiagen). The absence of protein or DNA contamination was confirmed according to *A*_260_/*A*_280_ ratios and standard PCR tests respectively. RNA was quantified using a NanoDrop spectrophotometer (NanoDrop Technologies) and integrity confirmed by formaldehyde agarose gel electrophoresis. Samples were snap-frozen in liquid nitrogen and stored at −20°C until use. Reverse transcription reactions were performed using Superscript III reverse transcriptase (Invitrogen) with 2 μg of RNA template per sample. Residual RNA was removed by treatment with *E. coli* RNase H (Invitrogen).

The relative abundance method was used to determine resultant cDNA levels by quantitative RT-PCR. Reactions (in triplicate) comprised 15 pmol each of gene-specific primers (HPLC purified, Sigma–Genosys), 2 μl cDNA template (10^−1^ dilution), 12.5 μl of 2× QuantiTect SYBR Green PCR Master Mix, made up to 25 μl with RNase-free water. PCRs [95°C for 15 min (95°C for 30 s, 52°C for 30 s, 72°C for 30 s) for 40 cycles] were monitored using a MX4000 RT-PCR thermocycler (Stratagene). Initial template concentrations were calculated using the MX4000 software, and results were normalized using *ACT1* as reference mRNA.

For semiquantitative RT-PCR, 1 μl of 10^−1^, 10^−2^, 10^−3^ and 10^−4^ dilutions of cDNA were used as templates in 50 μl PCRs [95°C for 5 min (95°C for 30 s, 52°C for 30 s, 72°C for 30 s) for 30 cycles; 72°C for 5 min] containing 50 pmol of each gene-specific primer. Red Hot Taq polymerase (AB Gene) was used for amplifications, and products were examined by agarose gel electrophoresis. The intensities of products at template dilutions in which the reaction had not progressed to saturation were estimated with ImageJ software (National Institutes of Mental Health, Maryland, USA) using *ACT1* as reference mRNA. All primers were designed using Primer 3 software (Rozen and Skaletsky, 2000).

### Western blotting

Preparation of *S. cerevisiae* lysates and Western blotting were performed using standard procedures (Ausubel *et al*., 2007). Proteins isolated from 2 × 10^6^ cells were separated by SDS-PAGE (10%) using an Invitrogen Nu-PAGE electrophoresis system, and blotted to PVDF membrane (Westram). Blots were probed with rabbit polyclonal anti-Gts1p primary antibody (a gift from Dr Kunio Tsurugi, Yamanashi Medical University) (1:2000 dilution), and alkaline phosphatase-conjugated anti-rabbit IgG secondary antibody (Sigma) (1:3000 dilution). Gts1p was detected with BCIP-NBT (Sigma) and quantified by densitometry with a Quantity One system (Bio-Rad).

### Flow cytometry, cell sorting and fluorescence microscopy

Samples of exponential-phase cells (2 × 10^7^ cells) in YPD were stained with the reduced-glutathione (GSH)-specific dye monochlorobimane (mBCl; Molecular Probes) (Stevenson *et al*., 2002) at 100 μg ml^−1^ for 10 or 15 min at 30°C. Cells were washed and resuspended in 2 ml PBS, briefly sonicated (Sanyo Soniprep 10 s, 2 μm), and analysed using a Coulter Epics Altra flow cytometer (Beckman) equipped with a UV laser. Data for fluorescence from mBCl via a 450 DF65 filter (PMT2) were collected for 50 000 cells in each sample. Cell-to-cell heterogeneity was determined as the CV [(standard deviation/mean) × 100%] after correction for autofluorescence using Weasel software (The Walter and Eliza Hall Institute of Medical Research, Australia). For Cd-resistance tests with culture subpopulations, cells were gated according to GSH content, sorted and spread plated onto YPD agar supplemented or not with Cd(NO_3_)_2_. Plates were incubated as described above to determine % viability. For analysis of GSH oscillations, sorted cells were incubated in YPD with shaking, and samples were removed at intervals for staining with mBCl and analysis as described above.

For fluorescence microscopy, cells were examined using a Zeiss Axioscope MS fluorescence microscope fitted with a HB050 illuminator (Carl Zeiss, Thornwood, NY), and images were captured with a Zeiss Axiocam digital camera fitted with a 470 DF20 filter.
